# Variation in tissue Na^+^ content and the activity of *SOS1* genes among two species and two related genera of Chrysanthemum

**DOI:** 10.1186/s12870-016-0781-9

**Published:** 2016-04-21

**Authors:** Jiaojiao Gao, Jing Sun, Peipei Cao, Liping Ren, Chen Liu, Sumei Chen, Fadi Chen, Jiafu Jiang

**Affiliations:** College of Horticulture, Nanjing Agricultural University, Nanjing, 210095 China

**Keywords:** Chrysanthemum morifolium, Compositae, SOS1, Functional characterization, Complementation assay

## Abstract

**Background:**

Chrysanthemum, a leading ornamental species, does not tolerate salinity stress, although some of its related species do. The current level of understanding regarding the mechanisms underlying salinity tolerance in this botanical group is still limited.

**Results:**

A comparison of the physiological responses to salinity stress was made between *Chrysanthemum morifolium* ‘Jinba’ and its more tolerant relatives *Crossostephium chinense*, *Artemisia japonica* and *Chrysanthemum crassum*. The stress induced a higher accumulation of Na^+^ and more reduction of K^+^ in *C. morifolium* than in *C. chinense*, *C. crassum* and *A. japonica*, which also showed higher K^+^/Na^+^ ratio. Homologs of an Na^+^/H^+^ antiporter (*SOS1*) were isolated from each species. The gene carried by the tolerant plants were more strongly induced by salt stress than those carried by the non-tolerant ones. When expressed heterologously, they also conferred a greater degree of tolerance to a yeast mutant lacking Na^+^-pumping ATPase and plasma membrane Na^+^/H^+^ antiporter activity. The data suggested that the products of *AjSOS1*, *CrcSOS1* and *CcSOS1* functioned more effectively as Na^*+*^ excluders than those of *CmSOS1*. Over expression of four *SOS1s* improves the salinity tolerance of transgenic plants and the overexpressing plants of *SOS1s* from salt tolerant plants were more tolerant than that from salt sensitive plants. In addition, the importance of certain AjSOS1 residues for effective ion transport activity and salinity tolerance was established by site-directed mutagenesis and heterologous expression in yeast.

**Conclusions:**

*AjSOS1*, *CrcSOS1* and *CcSOS1* have potential as transgenes for enhancing salinity tolerance. Some of the mutations identified here may offer opportunities to better understand the mechanistic basis of salinity tolerance in the chrysanthemum complex.

**Electronic supplementary material:**

The online version of this article (doi:10.1186/s12870-016-0781-9) contains supplementary material, which is available to authorized users.

## Background

Soil salinity is becoming a severe environmental stress all over the world. Currently, over 800 million hectares of the world’s arable land are adversely affected by salinity [1]. The major toxic cation present in saline soils is Na^+^, so under saline conditions, plants must minimize their cytosolic Na^+^ concentration to withstand the stress [2]. Three strategies have evolved to avoid the build-up of Na^+^ in the plant shoot: the first restricts the movement of the ion from the soil into the root, the second sequesters Na^+^ in the vacuole, and the third actively pumps Na^+^ out of the cytoplasm into the soil [1, 3–5]. Various ion transporters are involved in these processes, but a particularly prominent class is represented by the Na^+^/H^+^ antiporters. So far, two types of Na^+^/H^+^ antiporter NHE/NHX1 and NHA/SOS1 have been well characterized [2, 6].

*AtSOS1* is the first plasma membrane Na^+^/H^+^ antiporter gene cloned from higher plant, primarily expression of *AtSOS1* in epidermal cells at the root tip and in parenchyma at the xylem-symplast boundary of roots, stems and leaves, implying a role of this transporter in extruding Na^+^ to the growth medium and controlling long-distance Na^+^ transport in plants. Furthmore, under moderate salinity, *sos1* mutant accumulated less Na^+^ in its shoots than WT (wild-type) plants, also indicating that SOS1 participates in loading of Na^+^ into the xylem [7, 8]. Recently, several similar studies indicated this critical function in tomato [9] and in *Thellungiella salsuginea* [10]. SOS1 might also be involved in K^+^ nutrition in plants and under salt stress it is more vital for the plant to keep a high K^+^/Na^+^ ratio [6]. The *sos1* mutant showed significantly reduced high affinity K^+^ uptake and K^+^ content [11, 12], while higher K^+^ efflux from *sos1* root than that in WT plants [13]. Qi and Spalding (2004) demonstrated that SOS1 was required for protecting K^+^ uptake through AKT1 and compromised K^+^ nutrition during salt stress [14]. In addition, *ZxSOS1* controls long distance transport and spatial distribution of Na^+^ and K^+^ and maintains Na^+^, K^+^ homeostasis in the xerophyte *Zygophyllum xanthoxylum* [15]. Together, SOS1 is essential for plant to cope with salt stress by maintaining ions homeostasis and controlling long-distance Na^+^ transport via the xylem [16, 17].

The recognition of *AtSOS1* has facilitated the isolation of homologs from a growing number of plant species. Some of these have been tested by their heterologous expression in either yeast or bacterial hosts which lack their own Na^+^ transport system [9, 18–26]. Loss-of-function mutants of *AtSOS1* is salinity hypersensitive [12], while constitutive expression of *SOS1* in both *A. thaliana* itself as well as in other plant species, including chrysanthemum, improves the level of salinity tolerance [18, 20, 27–31].

The leading ornamental species chrysanthemum does not readily tolerate salinity stress, although some of its many related species do. The current level of understanding the mechanisms of salinity tolerance in this botanical group is still limited [32]. Here, the morphological effects of salinity stress, along with the extent of Na^+^ and K^+^ accumulation in chrysanthemum and its three more tolerant related species (*C. chinense, A. japonica* and *C. crassum*) have been explored. The *SOS1* homolog*s* present in each of the four species has been isolated and their contribution to salinity tolerance assessed by heterologously expressing them in a yeast mutant ANT3, and in transgenic chrysanthemum and *A. thaliana*. Furthermore, some important amino acid polymorphism for effective ion transport activity and salinity tolerance was also identified by mutagenesis.

## Results

### Variation for salinity tolerance in the chrysanthemum complex

**Fig. 1:**
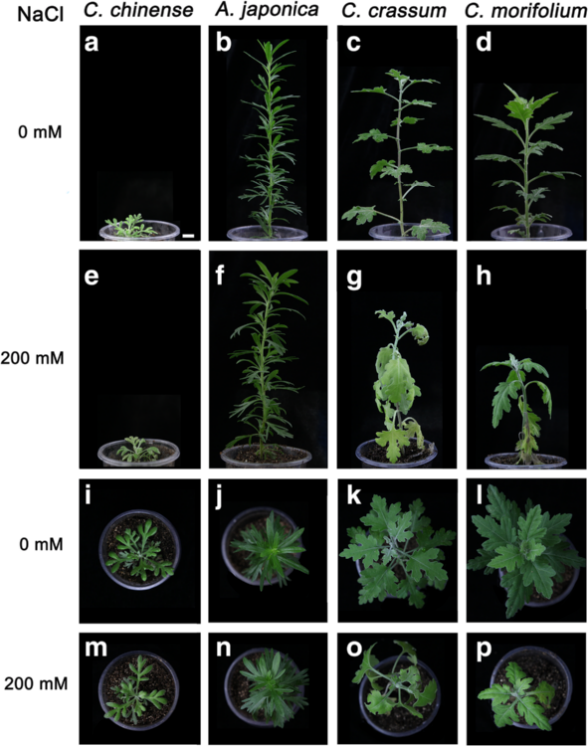
The phenotypic response of chrysanthemum and its three close relatives to a ten day exposure to 200 mM NaCl. **a**-**h** Side view; **i**-**p** Vertical view from above; **a**-**d** and **i**-**l** Plants grown in the absence of stress; **e**-**h** and **m**-**p** plants exposed to NaCl. **a**, **e**, **i**, **m** *C. chinense*, **b**, **f**, **j**, **n** *A. japonica*, **c**, **g**, **k**, **o** *C. crassum*, **d**, **h**, **l**, **p** *C. morifolium*. Bar = 1.0 cm

**Fig. 2:**
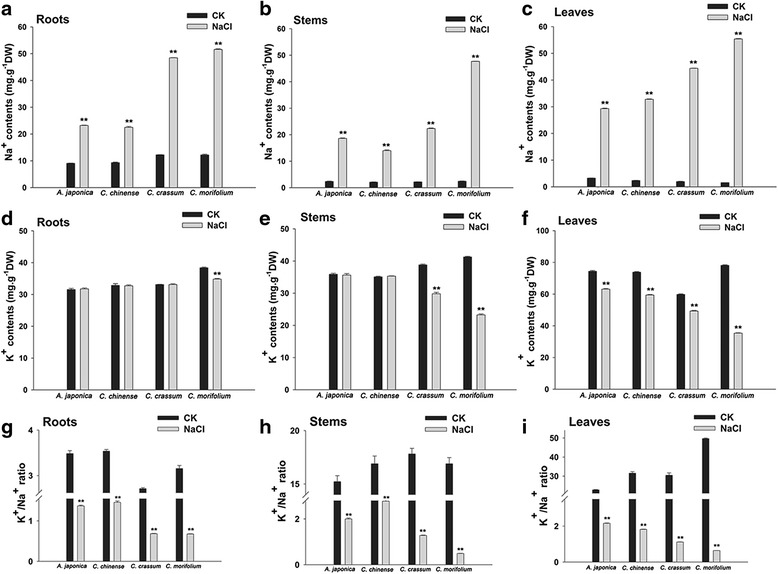
Variation in tissue Na^+^、K^+^ content and K^+^/Na^+^ ratio in the four test species in response to salinity stress. **a** Na^+^ content in the root, **b** Na^+^ content in the stem, **c** Na^+^ content in the leaf, **d** K^+^ content in the root, **e** K^+^ content in the stem, **f** K^+^ content in the leaf, **g** K^+^/Na^+^ ratio in the root, **h** K^+^/Na^+^ ratio in the stem, **i** K^+^/Na^+^ ratio in the leaf. *,**: means differ significant from levels in the control treatment (*P* < 0.05 and < 0.01, respectively)

Most of the leaves of *C. morifolium* plants became wilted and chlorotic following a ten day exposure to the salinity stress, and their lower leaves were largely necrotic *C. crassum* plants were less severely affected by the treatment, while there was no evidence of any damage to either *C. chinense* or *A. japonica* plants, the leaves of which stayed green, with the plants maintaining a near-normal level of growth for up to 14 days (Fig. 1). Under the non-stressed growing conditions, there was no variation in tissue Na^+^ concent between the four test species. However, when the plants were exposed to salinity, the tissue Na^+^ content throughout the plant was increased in all four species. The mean increase was notably lower for *C. chinense* and *A. japonica*: in these two species, the Na^+^ content in the roots (compared to the levels in non-stressed plants) rose by only 142.0 % and 156.0 % respectively, while for *C. crassum* and *C. morifolium* plants, the increase was 300.0 % and 324.0 % (Fig. 2a). The leaves behaved similarly, with the Na^+^ content rising more markedly in *C. morifolium* than in others. The Na^+^ content in the leaves of *C. morifolium* was 125.0 %, 169.0 % and 189.0 % that present in *C. crassum*, *C. chinense* and *A. japonica* plants exposed to the NaCl stress respectively (Fig. 2c). Moreover, the Na^+^ content in the stems behaved in a consistent way, it was highest in *C. morifolium*, moderate in *C. crassum* and low in both *A. japonica* and *C. chinense* (Fig. 2b).

K^+^ concent in the roots of *C. chinense*, *A. japonica* and *C. crassum* plants show nearly unchanged between control and salt stress except that of *C. morifolium* plants, whose K^+^ contents were significantly decreased (Fig. 2d). K^+^ level in both *A. japonica* and *C. chinense* stems was also unchanged, on the contrary, K^+^ content in the stems of *C. crassum* and *C. morifolium* was distinctly reduced by 23.2 % and 43.6 %, respectively (Fig. 2e). While K^+^ content in the leaves of all the plants tended to decrease, the reduction was far more significant in *C. morifolium*, i.e., 54.71 % (Fig. 2f). Relative to normal condition, salinity appreciably decreased the K^+^/Na^+^ ratio throughout the plants. *C. chinense* and *A. japonica* exhibited the highest K^+^/Na^+^ ratio of 5.7 and 5.6, respectively. In comparison, *C. morifolium* showed a minimum value in this ratio (1.8), while *C. crassum* showed an intermediate ratio of 3.1 (Fig. 2g-i). Overall, K^+^/Na^+^ ratio of the salt-sensitive plants was much lower than that of the salt-tolerant plants, indicating that salt-tolerant plants excluded Na^+^ and imported K^+^ more effectively than salt-sensitive plants did.

### Sequence analysis of the *SOS1* homologs

**Fig. 3:**
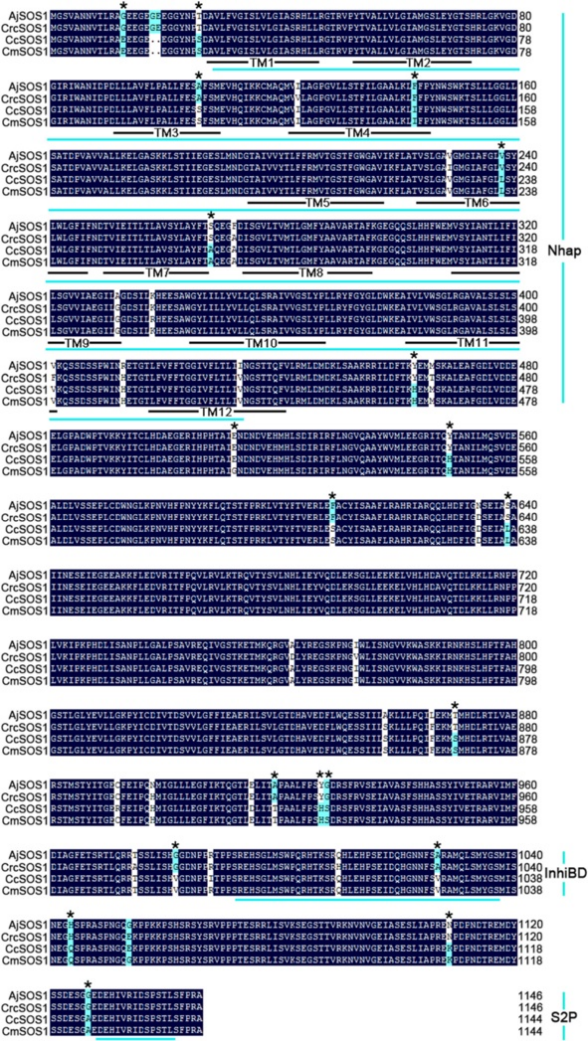
Multiple amino acid sequence alignment between four SOS1s. The 12 putative transmembrane domains are underlined and numbered 1 through 12. Residues conserved in at least two proteins are highlighted in white and blue. The black asterisks indicate conserved residues which were replaced in the site-directed mutagenesis experiment (see Additional file 2: Figure S4). Nhap, an Na^+^/H^+^ exchanger domain spanning the transmembrane region; InhiBD, an auto-inhibitory domain; S2P, SOS2 phosphorylation motif

**Fig. 4:**
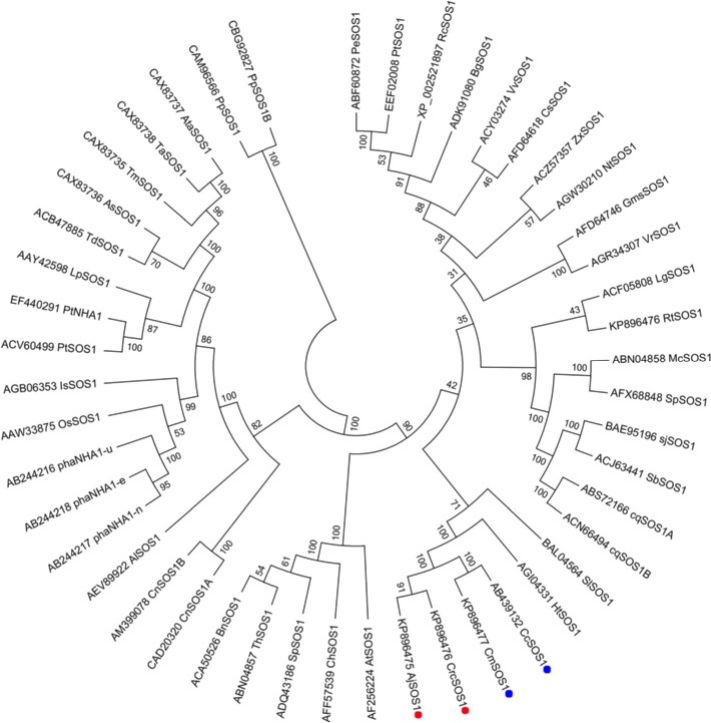
Phylogeny of the SOS1 proteins. *Artemisia japonic* AjSOS (KP896475), *Crossostephium chinense* CrcSOS1 (KP896476), *Chrysanthemum crissum* CcSOS1 (AB439132), *Chrysanthemum morifolium* CmSOS1 (KP896477), *Helianthus tuberosus* HtSOS1 (AGI04331), *Solanum lycopersicum* SlSOS1 (BAL04564), *Arabidopsis thaliana* AtSOS1 (AF256224), *Cochlearia hollandica* ChSOS1 (AFF57539), *Schrenkiella parvula* SpSOS1 (ADQ43186), *Eutrema halophilum* EhSOS1/ThSOS1 (ABN04857), *Brassica napus* BnSOS1 (ACA50526),*Glycine max* GmsSOS1 (AFD64746), *Vigna radiata* VrSOS1 (AGR34307), *Zygophyllum xanthoxylum* ZxSOS1 (ACZ57357), *Cucumis sativus* CsSOS1 (AFD64618), *Vitis vinifera* VvSOS1 (ACY03274), *Populus euphratica* PeSOS1 (ABF60872), *Bruguiera gymnorhiza* BgSOS1 (ADK91080), *Limonium gmelinii* LgSOS1 (ACF05808), *Mesembryanthemum crystallinum* McSOS1 (ABN04858), *Sesuvium portulacastrum* SpSOS1 (AFX68848), *Suaeda japonica* sjSOS1 (BAE95196), *Salicornia brachiata* SbSOS1 (ACJ63441), *Chenopodium quinoa* cqSOS1A (ABS72166); cqSOS1B (ACN66494), *Cymodocea nodosa* CnSOS1A (CAD20320); CnSOS1B (AM399078), *Aeluropus littoralis* AlSOS1 (AEV89922), *Phragmites australis* PhaNHA1-n (AB244217); PhaNHA1-e (AB244218); PhaNHA1-u (AB244216), *Oryza sativa* OsSOS1 (AAW33875), *Indosasa sinica* IsSOS1 (AGB06353), *Puccinellia tenuiflora* PtSOS1 (ACV60499), *Puccinellia tenuiflora* PtNHA1 (EF440291), *Lolium perenne* LpSOS1 (AAY42598), *Triticum durum* TdSOS1 (ACB47885), *Aegilops speltoides* AsSOS1 (CAX83736), *Triticum aestivum* TaSOS1 (CAX83738), *Aegilops tauschii* AtaSOS1 (CAX83737), *Triticum monococcum* TmSOS1 (CAX83735), *Physcomitrella patens* PpSOS1 (CAM96566); PpSOS1B (CBG92827), *Ricinus communis* RcSOS1 (XP_002521897), *Populus trichocarpa* PtSOS1 (EEF02008), *Nitraria tangutorum* NtSOS1 (AGW30210), *Reaumuria trigyna* RtSOS1 (AGW30208). The sequences were aligned using Clustal X and the phylogeny was constructed using the neighbor-joining method implemented in MEGA v5.0. The blue and red dots indicate the four *SOS1s* isolated here

A summary description of the *AjSOS1*, *CrcSOS1*, *CcSOS1* and *CmSOS1* sequences is given in Additional file 1: Table S2. The ORF (open reading frames) sequence of the derived *CcSOS1* fully matched that given in [23], but the UTR sequence differed slightly. All four SOS1 sequences were predicted to encode a Na^+^/H^+^ antiporter. Both AjSOS1 and CrcSOS1 harbored a 1147aa ORF, whereas the CcSOS1 and CmSOS1 products were two residues shorter. The secondary structure of the four SOS1 proteins featured 12 transmembrane domains in their N terminal region according to TMPRED and included a long hydrophilic cytoplasmic tail in their C terminal segment (Fig. 3). The levels of peptide identity between AjSOS1 and the other three proteins were 98.5 % (CrcSOS1), 97.0 % (CcSOS1) and 97.4 % (CmSOS1); those between CrcSOS1 and the other two proteins were 97.1 % (CcSOS1) and 97.3 % (CmSOS1); and that between CcSOS1 and CmSOS1 was 99.5 %. Comparisons with other plant SOS1s revealed a high degree of sequence conservation: for example the level of amino acid sequence identity between four cloned SOS1s and *A. thaliana* AtSOS1 was 90.3 %, with the tomato SlSOS1 91.8 %, with rice OsSOS1 90.1 % and with *Helianthus tuberosus* HtSOS1 94.7 %. A phylogenetic analysis between four SOS1s and other palnt SOS1 transports [7, 9, 15, 18, 19, 21, 24–27, 29, 33–46] showed that AjSOS1 and CrcSOS1 were closely relatives, as were CcSOS1 and CmSOS1, while the nearest relatives of the four SOS1s as a group were HtSOS1 and SlSOS1 (Fig. 4). The presence of three conserved domains is required for the activity and regulation of the SOS1 protein: these are Nhap (an Na^+^/H^+^ exchanger domain spanning the transmembrane region), InhiBD (an auto-inhibitory domain) and S2P (a phosphorylation motif recognized by SOS2) [19, 47], and all three were present in the four SOS1s analysed here (Fig. 3).

### *SOS1* transcription profiling

**Fig. 5:**
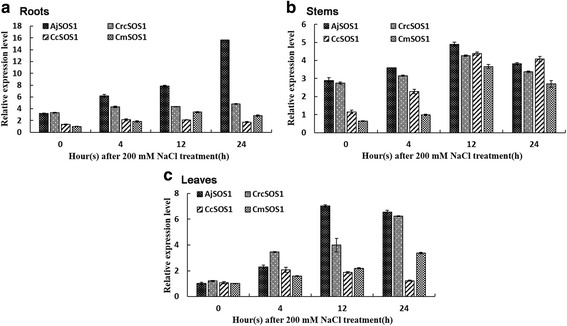
qPCR based transcription profiling of the four *SOS1* genes in response to salinity stress. Relative transcript abundances in the root (**a**), stem (**b**) and leaf (**c**). The relative expression in all tissues and time points was first compared to the reference genein each species and then calculated using the expression value at the initial time (0 h) in the root of *CmSOS1*. Data are presented as mean ± SE (*n* = 3). *Actin* was used as reference gene

In the roots, the abundance of *AjSOS1* transcript increased gradually of salinity stressed plants, reaching a level of 3.87 fold above the base level after a 24 h exposure to 200 mM NaCl. *CmSOS1* expression level increased only slowly over the first four hours of the treatment, peaking by 12 h, then decreased slightly, while the transcripts of *CrcSOS1* and *CcSOS1* maintained relatively constant (Fig. 5a). In the stems, all four *SOS1s* were up-regulated by the stress, their transcripts were greatest after 12 h (Fig. 5b). In the leaves, the level of transcription of both *CrcSOS1* and *CmSOS1* was highest at 24 h; that of *AjSOS1* rosed most sharply between 4 h and 12 h, thereafter declined; and that of *CcSOS1* remained relatively constant, with a two fold up-regulation occurring at 4 h (Fig. 5c). In essence, all four *SOS1s* were up-regulated by exposure to salinity, and the abundance of *SOS1* transcript was greater in the more salinity tolerant plants.

### Complementation of the yeast mutant with four *SOS1s*

**Fig. 6:**
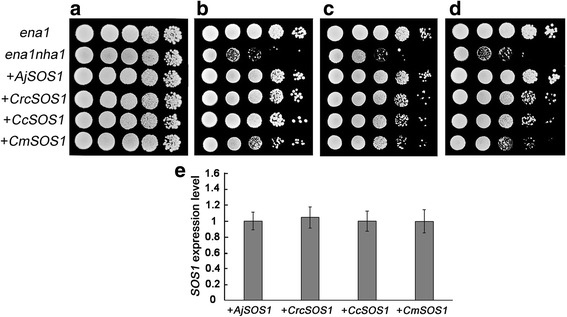
Functional characterization of four *SOS1s* in the salinity sensitive yeast mutant ANT3 (*ena1 nha1*) and expression analysis of *SOS1* gene in four *SOS1s* yeast transformants. ANT3 were transformed with plasmid containing four *SOS1s* (+*AjSOS1*, +*CrcSOS1*, +*CcSOS1*, +*CmSOS1*), G19 (*ena1*) and ANT3 (*ena1 nha1*) were transformed with the empty vector. G19 (*ena1*) cells were used as a positive control. Transformants were brought to a density 2 x 10^6^ per mL, of which 5 μL (serially diluted) were spotted onto YPDA medium containing 0 mM NaCl (**a**) and AP medium containing 30 (**b**), 50 (**c**) and 70 (**d**) mM NaCl. Plates were incubated at 30 °C for 2–4 days. **e** qPCR analysis of *SOS1* expression in four *SOS1s* yeast transformants. The *actin* gene was employed as an internal control

All the yeast cells grew freely on YPDA in the absence of NaCl and one of the four related *SOS1s* transformed ANT3 cells grew much better on AP NaCl-containing medium than the control strain (Fig. 6a-d). A comparison of the ability of four *SOS1s* transformants to grow in the presence of salt especially at 70 mM NaCl showed that the inclusion of *AjSOS1* was the most beneficial, followed by that of *CrcSOS1*; the strain carrying *CcSOS1* was better than *CmSOS1*, but was worse than *CrcSOS1*, while the inclusion of *CmSOS1* was the least salinity tolerant of the transformed cells (Fig. 6d). Furthermore, qPCR (quantitative real-time polymerase chain reaction) analysis of the *SOS1* expression levels were almost the same between yeast transformants for four *SOS1s* (Fig. 6e). The data demonstrated that Na^+^/H^+^ antiporter activity of four *SOS1s* was essential and *AjSOS1*, *CrcSOS1* and *CcSOS1* were fully able to exclude Na^+^ when expressed in yeast.

### Overexpression of four *SOS1s* enhances salinity tolerance in transgenic chrysanthemum and Arabidopsis plants

**Fig. 7:**
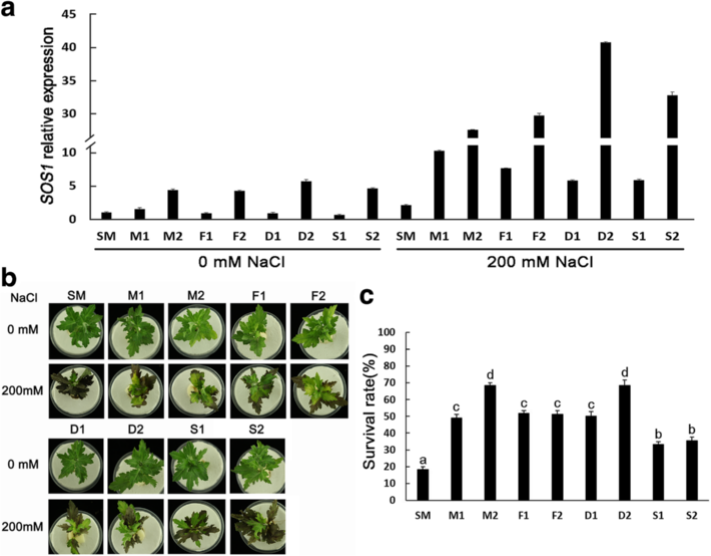
Salinity tolerance of wide type ‘Jinba’ and transgenic chrysanthemum plants overexpressing four *SOS1s*. **a** Expression levels of *SOS1* in wide type ‘Jinba’ and transgenic chrysanthemum lines overpressing four *SOS1s*. SM, wide type ‘Jinba’ plant; M1 and M2, transgenic chrysanthemum lines of *AjSOS1*; F1 and F2, transgenic chrysanthemum lines of *CrcSOS1*; D1 and D2, transgenic chrysanthemum lines of *CcSOS1*; S1 and S2, transgenic chrysanthemum lines of *CmSOS1*. **b** Phenotypic response of saline hydroponics with 200 mM NaCl for 3 days. **c** Plant survival measured at 4 day in the presence of saline hydroponics with 200 mM NaCl

Transgenic chrysanthemum lines overexpressing four *SOS1s* were successfully generated. qPCR analysis showed that compare with wide type (SM), *SOS1* transcript abundance was not very high in the eight transgenic lines under control conditions but increased greatly upon 200 mM NaCl treatment (Fig. 7a). When exposure to saline hydroponics, most of the apex and edge of the lower leaves of all plants showing signs of yellowing and necrotic after 1 day treatment, while after 3 days, the leaves of SM became severely necrotic and most plants died, the survival ratio of which was only 18 %. In the transgenic plants, symptoms of damage in leaves were much less evident in S1 and S2 than SM plants, but were worse than that of other transgenic plants, most of their upper leaves still remained green, and with less affected by salinity stress, which showed the transgenic plants maintained higher chlorophyll contents. The chlorophyll content is often used as index of salt tolerance in plants under salt stress, such as in *Arabidopsis* [48] and tobacco [49]. The percentage survival of S1 and S2 plants was 33 % and 36 %, respectively, whereas that of other transgenic plants was 49 %-68 % (Fig. 7b-c). Furthermore, each of two transgenic *A. thaliana* lines overexpressing four *SOS1s* were selected for further study. For example no expression of exogenous *SOS1* was detected in *A. thaliana* wide type *gl1* but in these transgenic lines M-1, M-2, F-1, F-2, D-1, D-2,S-1 and S-2, which showed a high expression level of *SOS1* (Additional file 3: Figure S2a). On 1/2 MS medium containing 150 or 75 mM NaCl, the seed germination rates, root length and fresh weight of transgenic *A. thaliana* wild type or *sos1-1* lines were higher than those of the corresponding, and in the transgenic lines, the above index value in gS-1, gS-2, sS-1 and sS-2 were also notably lower than that of other lines (Additional file 3: Figure S2 and Additional file 4: Figure S3). These results indicated that the differences between the *SOS1* transcription level in the transgenic lines did not very important effect in their tolerance to salinity, and over expression of four *SOS1s* enhanced the salinity tolerance of transgenic plants and the overexpressing plants of *SOS1s* from salt tolerant plants were more tolerant than *SOS1s* from salt sensitive plants.

### Site-Directed Mutagenesis functional analysis in yeast

**Fig. 8:**
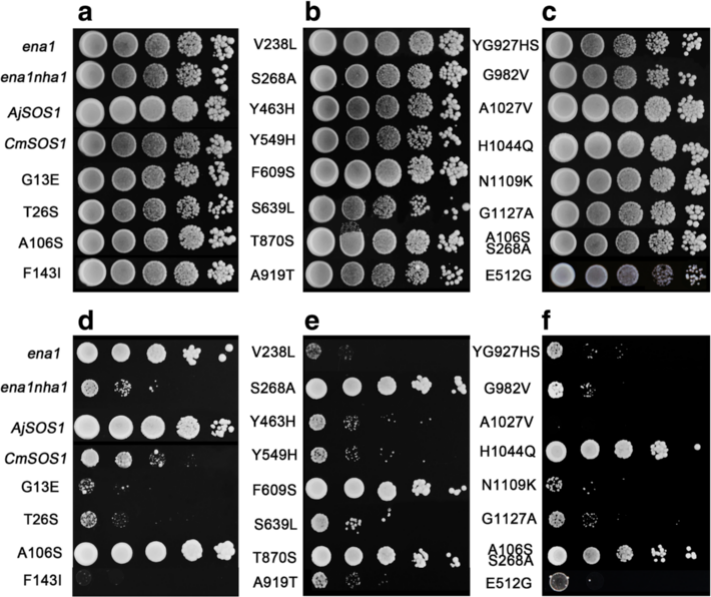
The salinity tolerance of ANT3 cells expressing altered forms of *AjSOS1*. The yeast cells were cultured overnight and a 5 μL aliquot (serially diluted) was spotted onto either a YPDA medium containing no NaCl (**a**-**c**) or an AP medium containing 70 mM NaCl (**d**-**e**). Plates were incubated at 30 °C for 2–4 days

The site-directed mutagenesis applied to *AjSOS1* produced a set of 18 residue polymorphisms and additional one site-directed mutagenesis applied to *CcSOS1* (Additional file 2: Figure S4). The hypothesis was that mutations a critical residue in *AjSOS1* or *CcSOS1* would generate a loss of salinity tolerance, as assayed by the yeast complementation test. The mutated forms were introduced into ANT3, and the drop test was conducted on AP medium containing 70 mM NaCl and 1 mM KCl. As depicted in Fig. 8, mutants G13E, T26S, F143I, V238L, Y463H, E512G, Y549H, S639L, A919T, YG927HS, G982V, A1027V, N1109K and G1127A failed to complement the growth defect of yeast cells, which suggested that these mutations couldn’t mediate Na^+^ efflux in yeast and may be important for transport activity and salt tolerance of *AjSOS1*. The other *AjSOS1*-mutants supported more cell growth than either empty vector transformed ANT3 cells or those transformed with *CmSOS1*, indicating that these mutants were null mutations.

## Discussion

At the phenotypic level, *C. chinense* and *A. japonica* both appeared to tolerate salinity stress rather better than either *C. crassum* or *C. morifolium* (Fig. 1), this finding consistent with the division of 32 chrysanthemum-related taxa into four clusters based on their morphological response to the stress [32]. The primary effect of salinity stress is a disturbance of cellular ion homeostasis, followed by the ingress of toxic levels of Na^+^ into the cytoplasm. Patterns of ion accumulation have been exploited with some success as a means of discriminating between tolerant and sensitive species/cultivars [50]. The present data showed that exposure to 200 mM NaCl induced a smaller increase in tissue Na^+^ content and a less reduction in tissue K^+^ content and K^+^/Na^+^ ratio in *C. chinense* and *A. japonica* than in *C. crassum* and *C. morifolium* (Fig. 2), consistent with the ranking based on the species’ morphological response. The main conclusion was that the variation in salt tolerance displayed by the four species most likely reflected genetic variation for their ability to exclude the ingress of Na^+^, most probably thanks to have a more selective ion transport system. Similar conclusions have been drawn from the study of a range of other plant species [51–54]. Na^+^ transporters are an important class of protein employed by *A. thaliana* to maintain ion homeostasis during an episode of salinity stress. The activity of *AtSOS1* is central to the exclusion of Na^+^, as well as to its loading and retrieval into and out of the xylem [8]. The existence of an efficient SOS pathway would therefore make a major contribution to the superior salinity stress tolerance of *C. chinense* and *A. japonica*.

The *SOS1* genes isolated from the four chrysanthemum and its related species all belong to the *A. thaliana* CPA1 (cation proton antiporter 1) family [6]. They all harbored three conserved functional domains Nhap, InhiBD and S2P (Fig. 3), a characteristic of *SOS1* encoded proteins, and thought to be critical for their functionality [47]. In the absence of salinity stress, the abundance of *SOS1* transcript in both the root and stem was higher in the more salinity tolerant *A. japonica* and *C. chinense* than in either *C. crassum* or *C. morifolium*, whereas in the leaf, the *SOS1* transcript abundance in the four species differed little. The *SOS1* genes were all up-regulated throughout the plant when salinity stress was imposed, inducing much higher transcript levels in the root than in either the stem or the leaf (Fig. 5). Moreover, the transcripts of reference gene *Actin* in four tested plants after salt treatment were relatively constant (Additional file 5: Figure S1). The behavior of *SOS1* genes in a range of glycophytes is quite similar [7, 10, 15, 18, 22, 29, 43, 55], although in other species, salinity stress has been found to significantly up-regulate *SOS1* in the leaf but not in root [26, 40, 42, 56]. In the former case, the assumption is that the SOS1 protein acts to remove Na^+^ from the root cell, while in the latter, they have been suggested to function as maintainers of a low cytosolic Na^+^ concentration in the leaf to protect photosynthesis. Notably, the abundance of *AjSOS1* and *CrcSOS1* transcript in salinity-stressed plants was greater than that of *CcSOS1* and *CmSOS1*, which concords with the differences in ion accumulation and salinity tolerance displayed by the four species. Similarly, in a contrast between the salinity tolerant *Populus euphratica* and the more sensitive *Populus popularis*, the former was seen to accumulate a higher transcript abundance of genes related to Na^+^/H^+^ antiporter activity [57]. Likewise, in a comparison of four *Brassica* spp. accessions, the more salinity tolerant entries displayed the highest level of *SOS1* transcription [53], while in bread wheat ‘Kharchia 65’, a cultivar known to be an efficient Na^+^ exporter also showed high levels of *SOS1* transcription [58]. Finally, in *A. thaliana,* the level of *SOS1* transcription in the root has been shown to be inversely proportional to the accumulation of Na^+^ in the plant [59]. Thus the evidence is very strong to support the notion that SOS1 proteins make an important contribution to salinity tolerance in the chrysanthemum species complex.

Heterologous expression in yeast has been exploited by a number of researchers aiming to functionally characterize plant *SOS1* genes [10, 18, 20–22, 25, 60]. The ANT3-based system effectively discriminated between the efficacy of the chrysanthemum and its related species *SOS1s* in terms of their ability to counteract salinity stress. In particular, the assay showed that the *AjSOS1* and *CrcSOS1* products were able to compensate for the yeast host’s lack of Na^+^-pumping ATPase ENA1-4 and plasma membrane Na^+^/H^+^ antiporter NHA1 activity and the *SOS1* expression levels of yeast transformants for four *SOS1s* were almost the same (Fig. 6). The implication is that these proteins mediate Na^+^ efflux at the plasma membrane of yeast. Since *AjSOS1*, *CrcSOS1* and *CcSOS1* were much more effective than *CmSOS1*, it seems probable that these proteins are key determinants of the contrasting ionic homeostasis and levels of salinity tolerance of the four species. Similar conclusions have been drawn by contrasting the effectiveness of an *SOS1* gene isolated from the salinity tolerant species *Thellungiella salsuginea* with that of *AtSOS1* [10], and that of the *SOS1* genes from the two halophytes *Eutrema salsugineum* and *Schrenkiella parvula* [35]. Takahashi et al. (2009) have shown that yeast cells heterologously expressing a *PhaNHA1* allele (*PhaNHA1-n*) isolated from a salinity tolerant reed plants grew better than those harboring an allele (*PhaNHA1-u*) isolated from a salinity sensitive accession [21].

Several researchs have been shown that transgenic plants over-expression *SOS1* improved salt tolerance [18, 20, 27–31, 61]. In this study, we demonstrated that over expression of four *SOS1s* also enhanced the salinity tolerance of transgenic chrysanthemum and *A. thaliana* wild type or *sos1-1*, and the overexpressing plants of *SOS1s* from salt tolerant plants were more tolerant than that from salt sensitive plants (Fig. 7, Additional file 3: Figure S2 and Additional file 4: Figure S3). These results were consist with the above functional analysis in the yeast mutant. To understand the reason for the different activities at *SOS1s*, a multiple alignment of four SOS1s proteins was analyzed and found that the AjSOS1 and CrcSOS1 sequences differed from CcSOS1 and CmSOS1 with respect to eighteen residues, and additional one residues in which CmSOS1 encode amino acid relative to the same ones of the other three SOS1s, and of which six were located in the membrane-spanning region and the other thirteen in the hydrophilic tail (Fig. 3).

When site-directed mutagenesis was carried out, it was found that a number of the altered polypeptides had no deleterious effect on the ability to complement the lesion in the ANT3 cell line, showing that these residues were not determinants of the protein’s functionality. However, some of the altered polypeptides (G13E, T26S, F143I, V238L, Y463H, E512G, Y549H, S639L, A919T, YG927HS, G982V, A1027V, N1109K and G1127A) did reduce the level of the yeast’s salinity tolerance, implying that these were essential for endowing *AjSOS1* with the capacity to compensate for the host’s defective Na^+^-pumping ATPase and plasma membrane Na^+^/H^+^ antiporter activity (Fig. 8). G13E and T26S lie at the 5′ end of TMD1, F143I and V238L in the TMD4 and TMD6 respectively, while the remaining sites map to the C terminal hydropholic tail. Transmembrane regions in plant NHAs are thought to be important for Na^+^ and H^+^ exchange. The presence of a cytoplasmic tail indicates that the transporter is probably regulated by an external signal: under either salinity or oxidative stress, the AtSOS1 cytoplasmic tail interacts with RCD1, a regulator of the oxidative stress response [62]. Therefore, it is possible that the differential activity of the four SOS1s reflects a dissimilar interaction between their cytoplasmic tail and a signaling protein such as RCD1. Some of the mutations to AjSOS1 are likely to have induced alterations to the protein’s secondary structure (Additional file 6: Figure S5), thereby potentially affecting its regulation and functionality. In *A. thaliana*, the salinity sensitive mutations *sos1-3*, *sos1-8*, *sos1-9* and *sos1-12* each comprise a single residue substitution in AtSOS1 [7], while the substitution E1044V in the putative auto inhibitory domain of *E. salsugineum EsSOS1* is necessary, but not sufficient to facilitate the growth of AXT3K (Δ*ena1::HIS3::ena4*, Δ*nha1::LEU2*, Δ*nhx1::KanMX4*) yeast cells cultured on a saline medium [35]. In *Triticum durum,* the mutation of TdSOS1 alleles S1126A and S1128A (DSPS mutated to DAPA) have been associated with a reduced phosphorylation ability by the *A. thaliana* SOS2 kinase T/DSOS2Δ308, thereby preventing its activation of TdSOS1 [19]. Furthermore the alleles AtSOS1 S1136A and S1138A both interfere with phosphorylation by SOS2, while the G777D variant (*sos1-8*) is not activated by SOS2 [47]. Further investigations will be needed to provide much more evidences for contribution of the four *SOS1s* homologs in salt tolerance and to understand the basis of the observed variation in the activity of the *AjSOS1* alleles.

## Conclusions

In summary, in the four chrysanthemum and its related species, *C. chinense*, *A. japonica* and *C. crassum* were better tolerate than *C. morifolium.* They also had a superior capacity to prevent the accumulation of Na^+^ and the reduction of K^+^ in planta and their level of *SOS1* transcription was higher. Moreover *SOS1* sequence polymorphisms may be responsible for the higher efficacy of the *AjSOS1* encoded protein. Taken together, *AjSOS1*, *CrcSOS1* and *CcSOS1* might be potential genes for enhancing salinity tolerance through transgenic strategies.

## Methods

### Plant materials, growing conditions and the assessment of salinity tolerance

Samples of *C. chinense*, *A. japonica*, *C. crassum* and *C. morifolium* were obtained from the Chrysanthemum Germplasm Resource Preserving Centre (Nanjing Agricultural University, China). Uniform cuttings were vegetatively propagated in sand. For experiments designed to estimate Na^+^ and K^+^ content under salinity stress, a set of rooted seedlings at the 6–10 leaf stage \was transplanted into a 1:1 mixture of garden soil and vermiculite, and the plants cultured under a 16 h photoperiod, a day/night temperature of 22 °C/18 °C and a relative humidity of 68–75 %. The plants were irrigated with 200 mM NaCl every four days and photographed on day 10. Leaves, stems and roots were harvested separately on day 14, baked at 80 °C for three days and weighed. A 0.1 g aliquot of each dry sample was digested in 2 mL 10 M HNO_3_, after which the solution were added to 10 mL with distilled water. Na^+^ and K^+^ contents were measured in this extract using an Optima 2100DV inductively coupled plasma optical emission spectrometer (Perkin Elmer, USA) [63]. Each sample was replicated three times. Means were compared using the Student’s *t* test implemented in SPSS v17.0 J software (SPSS Inc., Chicago, IL, USA).

### Isolation of *SOS1* sequences

RNA (ribonucleic acid) was extracted from roots of plants exposed to a hydroponic solution (half strength Hoagland’s solution) containing 200 mM NaCl using the RNAiso reagent (TaKaRa Bio, Tokyo, Japan), following the manufacturer’s protocol. A 1 μg aliquot of this RNA was reverse transcribed via M-MLV reverse transcriptase (TaKaRa Bio), primed by Oligo d(T)_18_. The *SOS1* coding regions were amplified from the first strand cDNA (complementary deoxyribonucleic acid) using fusion PCR/overlap PCR [64] and the primer pairs F1/R1 and F2/R2 (sequences given in Table 1). The full length cDNA sequences were deduced from 5′ and 3′ RACE (rapid amplification of cDNA ends) amplicons [65], and then amplified from cDNA template using the primer pair Full-F/R (sequences given in Table 1). These amplicons were inserted into the pEASY-Blunt Zero Cloning vector (TransGen Biotech, Beijing, China) for sequence-based validation. Open reading frames (ORFs) was identified using the ORF finder program (www.ncbi.nlm.nih.gov/gorf/gorf.html). Hydrophobicity and putative transmembrane domain were predicted using the TMPRED program (www.ch.embnet.org/software/TMPRED_form.html). Multiple peptide alignment and phylogenetic analysis were carried out by DNAman v5.2.2.0 software (Lynnon Biosoft, St Louis, Canada), Clustal X and MEGA v5.0, which utilized the Neighbor-Joining method.

**Table 1:** Adaptor and PCR primer sequences

Primer name	5′–3′ sequence	Usage
F1	ATGGGATCGGTGGCAAACAAC	overlap ORF fragment1
R1	GCTTGTTTGCAGAAACTTGT	overlap ORF fragment1
F2	ATTTCTAAATGGTGTGCAAGC	overlap ORF fragment2
R2	TTAGGGAGCTCGGGGGAAAG	overlap ORF fragment2
Oligo d (T)_18_	TTTTTTTTTTTTTTTTTT	Reverse transcription
AAP	GGCCACGCGTCGACTAGTACGGGIIGGGIIGGGIIG	5′ -RACE
AUAP	GGCCACGCGTCGACTAGTAC	5′ -RACE
GSP5′-1	CTCTAAGCAAATGTCTTG	5′ -RACE
GSP5′-2	TCCTCCTCCGCCCTTAG	5′ -RACE
GSP5′-3	GTTTGCCACCGATCCCAT	5′ -RACE
Adaptor J-T	CTGATCTAGAGGTACCGGATCCTTTTTTTTTTTTTTTTT	3′ -RACE
Adaptor J-R	CTGATCTAGAGGTACCGGATCC	3′ -RACE
GSP3'-1	TCAGAAGGTTCTACGACAGTGAG	3′ -RACE
GSP3′-2	CCAGACCCAAATGATACTCGTGA	3′ -RACE
GSP3′-3	TACACTATCTTTCCCCCGAGCTC	3′ -RACE
Full-F	TGGTGGAGATGGGATCGGTGGCA	ORF amplifications
Full-R	CTAGTAAATATATTATACAAGTC	ORF amplifications
SOS1s-Sal-F	GCGTCGACATGGGATCGGTGGCAAACAACGTG	Functional complementation
SOS1s-Not-R	TTGCGGCCGCGATTAGGGAGCTCGGGGGAAAG	Functional complementation
Actin-F	AGCTTGCATATGTTGCTCTTGA	qPCR
Actin-R	TTACCGTAAAGGTCCTTCCTGA	qPCR
DL-F	TGGAGCTGAGGATGAACA	qPCR
DL-R	CTACCGTACTTTCTATGAACAC	qPCR
actin-F	GTGATGTCGATGTCCGTAA	qPCR
actin-R	AGAAGCCAAGATAGAACCA	qPCR
CmEF1α-F	TTTTGGTATCTGGTCCTGGAG	qPCR
CmEF1α-R	CCATTCAAGCGACAGACTCA	qPCR
AtAct2-F	TTCGTTTTGCGTTTTAGTCCC	RT-PCR
AtAct2-R	GGGAACAAAAGGAATAAAGAGGC	RT-PCR

### *SOS1* transcription profiling

Leaf, stem and root samples from four plants per species were collected at 0, 4, 12 and 24 h following the addition of 200 mM NaCl to the hydroponic solution, snap-frozen in liquid nitrogen, and used as a source of cDNA to provide the template for a qPCR assay. The necessary RNA extraction and reverse transcription were performed as described above. The primer pair DL-F/R (sequences given in Table 1) were designed to amplify *SOS1* fragment. The *Actin* gene (GenBank accession number AB205087) was used as the reference sequence. Each 20 μL reaction contained 10 μL SYBR Premix Ex Taq^TM^ II (TaKaRa Bio), 10 ng cDNA and 0.2 μM of each primer. The amplification program comprised an initial denaturation (95 °C/2 min), followed by 40 cycles of 95 °C/15 s, 55 °C/15 s and 72 °C/20 s. Relative transcript abundances were estimated using the 2^−ΔΔ*C*t^ method in compliance with MIQE guidelines [66, 67], and normalized against the transcript abundance at 0 h in the root of *CmSOS1* at the respective time-point.

### Complementation by the four *SOS1* genes in the yeast mutants ANT3

The two bakers’ yeast (*Saccharomyces cerevisiae*) mutant strains G19 (Δ*ena1::HIS3::ena4*) and ANT3 (Δ*ena1::HIS3::ena4*, Δ*nha1::LEU2*) were employed for performing a complementation assay. The former mutant lacks the Na^+^-pumping ATPase ENA1 to ENA4 while the latter,both the Na^+^-pumping ATPase and the plasma membrane Na^+^/H^+^ antiporter NHA1 are defective [8, 68]. First, the ORF of four *SOS1s* were amplified using Phusion High Fidelity DNA Polymerase (Thermo Scientific, USA) with the primer pair SOS1s-Sal-F/Not-R (sequences given in Table 1). Both the resulting amplicons and pENTR^TM^1A plasmid were digested with *Sal* I and *Not* I restriction sites and the corresponding bands were ligated to yield the entry vector pENTR^TM^1A-*SOS1s*. Then the pENTR^TM^1A vector containing *SOS1s* were inserted into the yeast expression vector pAD426GPD [69] using LR Clonase II enzyme mix (Invitrogen, USA). The pAD426GPD-*SOS1s* constructs were validated by sequencing, then transformed into ANT3 cells, utilizing the Yeastmaker transformation system 2 (Clontech, Mountain View, CA, USA). The empty vector pAD426GPD was transformed into strain ANT3 and G19, which used as positive control. Transformants were selected by culturing on SD standard medium lacking uracil. The yeast cells’ salinity tolerance phenotype was explored using a drop test in which a 5 μL aliquot of a saturated yeast culture, along with a similar volume of a serial dilution, was spotted onto alkali cation-free AP plates containing 1 mM KCl and with NaCl as designated [68, 70]. The plates were held at 30 °C for 2–4 days before being photographed. Moreover, four *SOS1s* transformants were cultured overnight and extracted RNA by using a fungi RNA extraction kit (Huayueyang biotech, Beijing, China). The reverse transcription and qPCR were performed as described above. The gene specific primer pair were DL-F/R and the reference gene was *actin* (GenBank accession number AAA34391), primer sequences were given in Table 1.

### Generation of four *SOS1s* over-expressors and their response to salinity treatment

To further analyze the function of the four *SOS1s* and confirm their importance in plant salt tolerance, the plasmid pENTR^TM^1A-*SOS1s* were subjected to the LR reaction to obtain expression vector pMDC32-*SOS1s* and introduced the constructs p2 × 35S:: *SOS1s* into salt sensitive *C. morifolium* ‘Jinba’, *A. thaliana* (Columbia ecotype) wide type *gl1* and mutant *sos1-1* via *Agrobacterium tumefaciens* strain EHA105 mediated leaf disc and floral pollen dip method as described above [71]. RNA was isolated from control and 200 mM NaCl treated putative transgenic chrysanthemum and wide type (SM) plants, and processed for qPCR directed to *SOS1* (using the primer pair DL-F/R) as described above. The primer pair CmEF1α-F/R was used to amplify the reference gene *CmEF1α*. Relative gene expression levels were also estimated using the 2^−ΔΔ*C*t^ method [66], and normalized against the expression level of *SOS1* in wide type plants under control conditions. Twenty plants of each transgenic lines (M1, M2, F1, F2, D1, D2, S1 and S2) and SM plants with three replicates were exposed to either control and a liquid nutrient solution (half strength Hoagland’s solution) supplemented with 200 mM NaCl conditions, photographed on day 3 and calculated the survival rate on day 4. In addition, putative Arabidopsis transformants were firstly screened on hygromycin medium and then identified by RT-PCR analysis, based on the primer pair DL-F/R. Transgenic lines were used to assess salinity tolerance on 1/2 MS (Murashige and Skoog) agar medium supplemented with NaCl, as indicted for each case. Each experiment was performed three times and significant differences among treatments were identified by one-way analysis of variance and Tukey’s multiple range test (*p* = 0.05). All statistical analyses were performed using SPSS v17.0 J software (SPSS Inc).

### Site-directed mutagenesis and the efficacy of mutated forms of *AjSOS1*

PCR-based site-directed mutagenesis was applied to the pENTR^TM^1A-*AjSOS1* construct using Pfusion High Fidelity DNA Polymerase (Thermo Scientific), based on the primers TB1F/R-TB18F/R according to manual of Quickchange® Site-Directed Mutagenesis Kit (Stratagene, La Jolla, CA, USA). In addition, we also produced another one mutations which was carried out on pENTR^TM^1A-*CcSOS1* plasmid DNA and based on the primer TB19F/R. All the mutated primer sequences were given in Additional file 7: Table S1. Each 50 μL reaction contained 5 μL 10 × reaction buffer, 1 μL 10 mM dNTP, 2 μL of each primer (10 μM/L), 1 μL Pfu DNA Polymerase (2.5 U/μL) (Thermo Scientific), 2 μL plasmid DNA (5 ng/μl) and 37 μL ddH_2_O. The reactions were initially denatured (95 °C/30 s), then subjected to 16 cycles of 95 °C/30 s, 55 °C/60 s and 72 °C/7 min. The resulting amplicons were digested with *Dpn* I and then inserted into *E. coli DH5α* competent cells. All plasmid constructs were sequenced to ensure that no unexpected mutations or cloning errors had occurred. The mutated constructs were then recombined into pAD426GPD and transformed in mutant ANT3 to assay the cells’ salinity tolerance phenotype, as described above.

### Plant line abbreviations

M1 and M2 (Transgenic chrysanthemum lines of *AjSOS1*), F1 and F2 (Transgenic chrysanthemum lines of *CrcSOS1*), D1 and D2 (Transgenic chrysanthemum lines of *CcSOS1*), S1 and S2 (Transgenic chrysanthemum lines of *CmSOS1*), SM (Wide type ‘Jinba’ plant).

gM-1 and gM-2 (Transgenic lines of *AjSOS1* in *A. thaliana* wide type *gl1*), gF-1 and gF-2 (Transgenic lines of *CrcSOS1* in *A. thaliana* wide type *gl1*), gD-1 and gD-2 (Transgenic lines of *CcSOS1* in *A. thaliana* wide type *gl1*), gS1 and gS2 (Transgenic lines of *CmSOS1* in *A. thaliana* wide type *gl1*).

sM-1 and sM-2 (Transgenic lines of *AjSOS1* in *A. thaliana* mutant *sos1-1*), sF-1 and sF-2 (Transgenic lines of *CrcSOS1* in *A. thaliana* mutant *sos1-1*), sD-1 and sD-2 (Transgenic lines of *CcSOS1* in *A. thaliana* mutant *sos1-1*), sS1 and sS2 (Transgenic lines of *CmSOS1* in *A. thaliana* mutant *sos1-1*).

### Ethics (and consent to participate)

Not applicable.

### Consent to publish

Not applicable.

### Availability of data and materials

The data sets supporting the results of this article are included within the article and its additional files.

## Additional files

Additional file 1: Table S2. Summary details of the four *SOS1* sequences isolated from chrysanthemum and its close relatives*. (DOCX 14 kb)*
Additional file 2: Figure S4. The site-directed amino acid in AjSOS1 secondary structure as predicted by TMPRED. (TIF 574 kb) Additional file 3: Figure S2. Salt tolerance phenotypes of transgenic A. thaliana wide type gl1 lines. (a) RT-PCR analysis of SOS1 in transgenic lines and wide type gl1. (b) Seeds of wide type gl1 and the four SOS1stransgenic lines (gM-1, gM-2, gF-1, gF-2, gD-1, gD-2, gS-1 and gS-2) were germinated directly on 1/2 MS medium and on 1/2 MS mediumsupplemented with 150 mM NaCl, and then grown for 7 days. (c) Six-day-old seedlings of gl1 and eight transgenic lines (gM-1, gM-2, gF-1, gF-2, gD-1, gD-2, gS-1 and gS-2) were transferred to 1/2 MS medium containing 150 mM NaCl. The pictures were taken sfter 14 days of treatment. seedling primary root lenth (d) and fresh weight (e) were measured at day 14 after transfer. Error bars represent SD (n = 15). (TIF 7669 kb) Additional file 4: Figure S3. Functional complementation of Arabidopsis mutant sos1-1 by four SOS1s. (a) Germination of sos1-1 and eight transgenic Arabidopsis mutant sos1-1 lines (sM-1, sM-2, sF-1, sF-2, sD-1, sD-2, sS-1 and sS-2) after 7 days sown on 1/2 MS and 1/2 MS with 75 mM NaCl. (b) Mutant sos1-1 and four SOS1s transgenic seedlings grown on 1/2 MS medium for six days and then transferred to 1/2 MS medium containing 75 mM NaCl and imaged after 7 days of salt treatment and the root elongation (c) and fresh weight of each of above mentioned lines were measured. Error bars represent SD (n = 15). (TIF 1979 kb) Additional file 5: Figure S1. Amplification curves (a) and melting curve analysis (b) of reference gene *Actin* in four tested plants after salt treatment. (TIF 2714 kb) Additional file 6: Figure S5. The predicted secondary structure of AjSOS1 prior to and after site-directed mutagenesis by DNAStar software. (TIF 2480 kb) Additional file 7: Table S1. Oligonucleotide sequences used for the site-directed mutagenesis. (DOCX 15 kb)

## Abbreviations

cDNA: complementary deoxyribonucleic acid
MS: Murashige and Skoog
ORF: open reading frames
qPCR: quantitative real-time polymerase chain reaction
RACE: rapid amplification of cDNA ends
RNA: ribonucleic acid
WT: wide type
