# Supplementary material for: Variation in tissue Na+ content and the activity of SOS1 genes among two species and two related genera of Chrysanthemum
Source: BMC Plant Biol. 2016 Apr 21;16:98. doi: 10.1186/s12870-016-0781-9 (PMC4839091; doi:10.1186/s12870-016-0781-9)
Supplement: Additional file 1: Table S2. — Summary details of the four SOS1 sequences isolated from chrysanthemum and its close relatives. (DOCX 14 kb) [file 12870_2016_781_MOESM1_ESM.docx]

| Name of cloned *SOS1s* | 3'UTR  (bp) | ORF  (aa) | 5'UTR  (bp) | Full length  cDNA(bp) | accession  number | molecular mass  （kDa） | Isoelectric  point |
| --- | --- | --- | --- | --- | --- | --- | --- |
| *AjSOS1* | 138 | 1147 | 229 | 3811 | KP896475 | 127.06 | 6.52 |
| *CrcSOS1* | 115 | 1147 | 223 | 3782 | KP896476 | 127.11 | 6.40 |
| *CcSOS1* | 106 | 1145 | 224 | 3768 | AB439132 | 126.98 | 6.52 |
| *CmSOS1* | 111 | 1145 | 226 | 3773 | KP896477 | 126.81 | 6.60 |
