# Supplementary figures and images for: Variation in tissue Na+ content and the activity of SOS1 genes among two species and two related genera of Chrysanthemum
Source: BMC Plant Biol. 2016 Apr 21;16:98. doi: 10.1186/s12870-016-0781-9 (PMC4839091; doi:10.1186/s12870-016-0781-9)

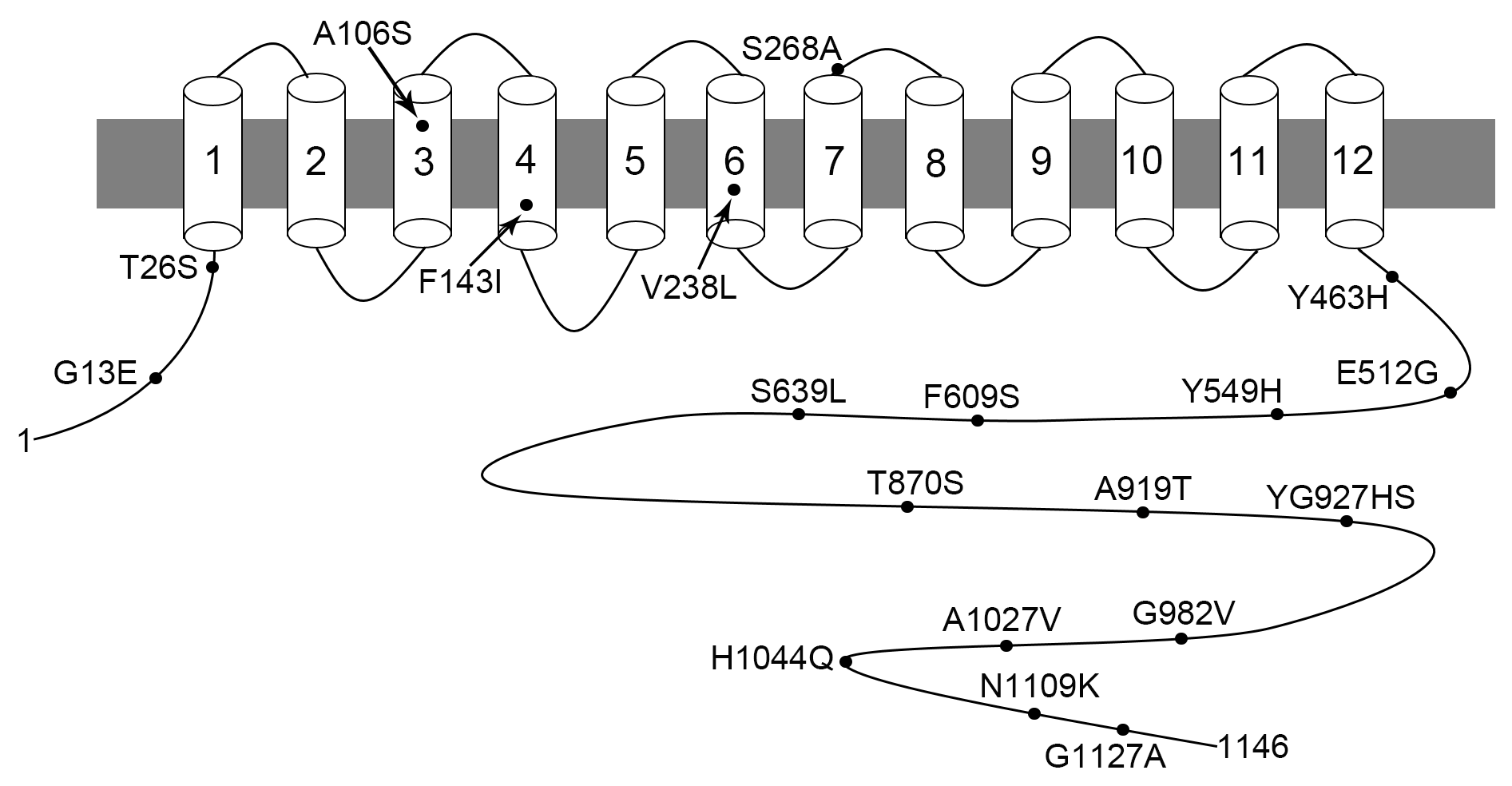

Supplement: Additional file 2: Figure S4. — The site-directed amino acid in AjSOS1 secondary structure as predicted by TMPRED. (TIF 574 kb) [file 12870_2016_781_MOESM2_ESM.tif]

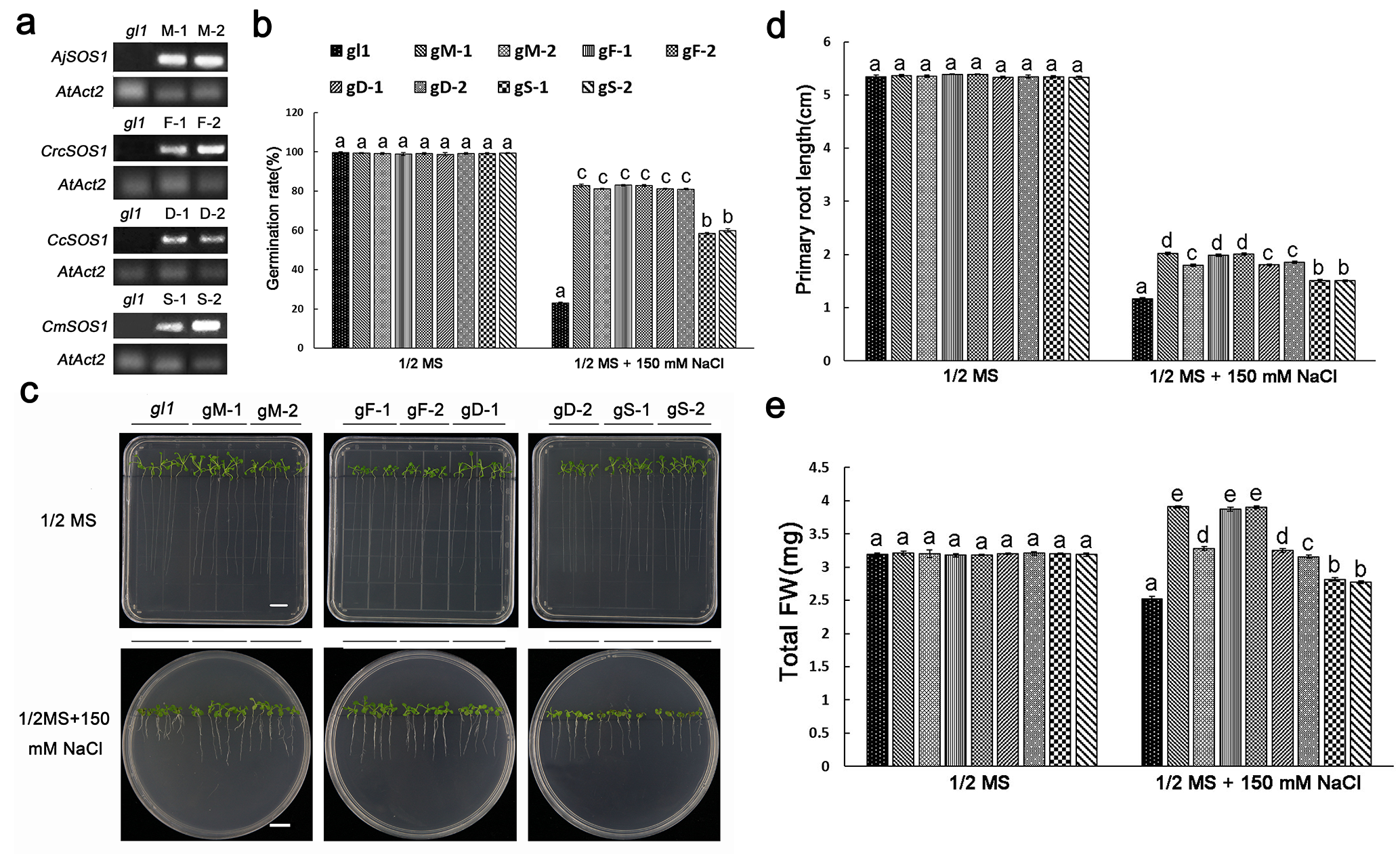

Supplement: Additional file 3: Figure S2. — Salt tolerance phenotypes of transgenic A. thaliana wide type gl1 lines. (a) RT-PCR analysis of SOS1 in transgenic lines and wide type gl1. (b) Seeds of wide type gl1 and the four SOS1stransgenic lines (gM-1, gM-2, gF-1, gF-2, gD-1, gD-2, gS-1 and gS-2) were germinated directly on 1/2 MS medium and on 1/2 MS mediumsupplemented with 150 mM NaCl, and then grown for 7 days. (c) Six-day-old seedlings of gl1 and eight transgenic lines (gM-1, gM-2, gF-1, gF-2, gD-1, gD-2, gS-1 and gS-2) were transferred to 1/2 MS medium containing 150 mM NaCl. The pictures were taken sfter 14 days of treatment. seedling primary root lenth (d) and fresh weight (e) were measured at day 14 after transfer. Error bars represent SD (n = 15). (TIF 7669 kb) [file 12870_2016_781_MOESM3_ESM.tif]

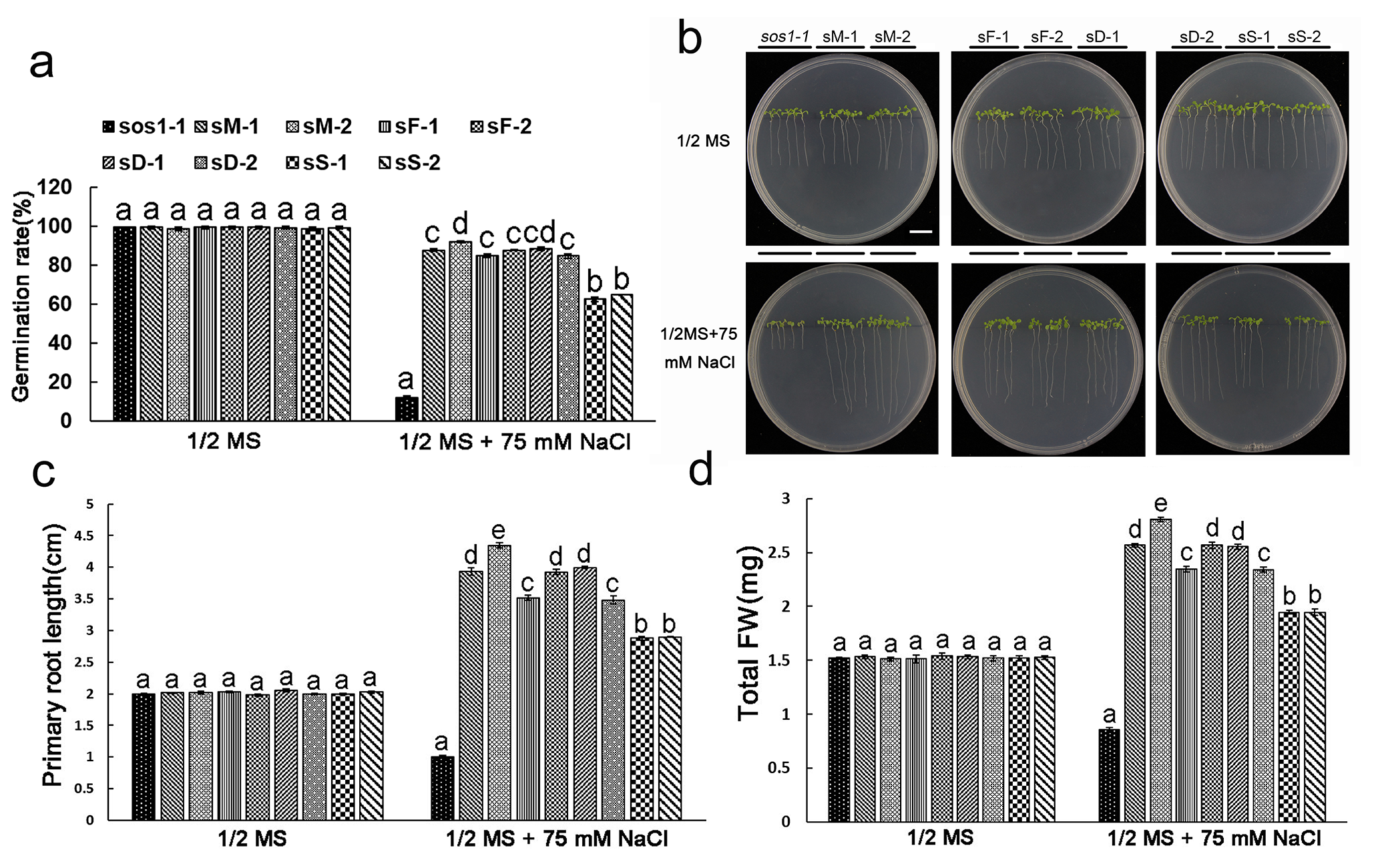

Supplement: Additional file 4: Figure S3. — Functional complementation of Arabidopsis mutant sos1-1 by four SOS1s. (a) Germination of sos1-1 and eight transgenic Arabidopsis mutant sos1-1 lines (sM-1, sM-2, sF-1, sF-2, sD-1, sD-2, sS-1 and sS-2) after 7 days sown on 1/2 MS and 1/2 MS with 75 mM NaCl. (b) Mutant sos1-1 and four SOS1s transgenic seedlings grown on 1/2 MS medium for six days and then transferred to 1/2 MS medium containing 75 mM NaCl and imaged after 7 days of salt treatment and the root elongation (c) and fresh weight of each of above mentioned lines were measured. Error bars represent SD (n = 15). (TIF 1979 kb) [file 12870_2016_781_MOESM4_ESM.tif]

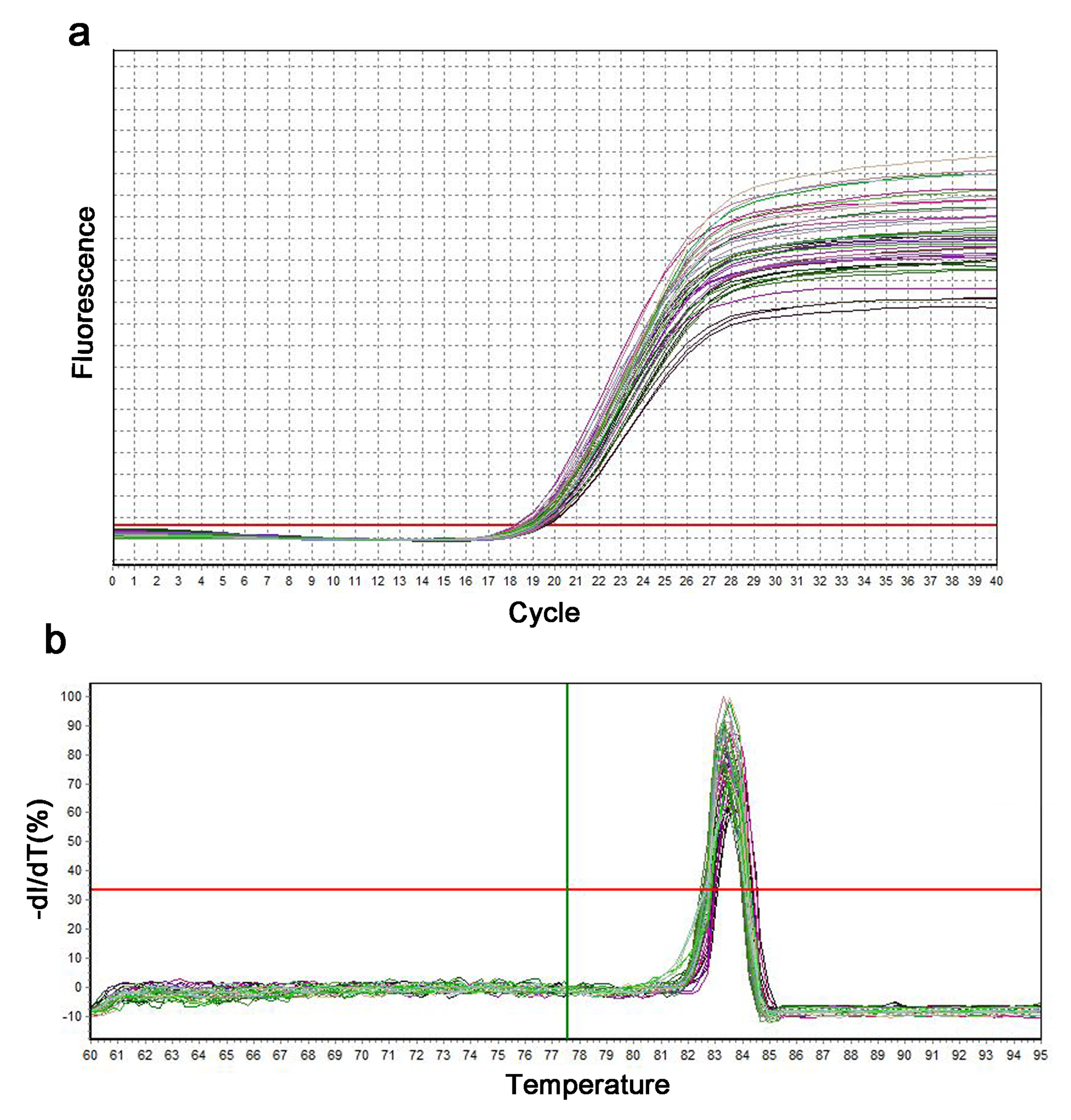

Supplement: Additional file 5: Figure S1. — Amplification curves (a) and melting curve analysis (b) of reference gene Actin in four tested plants after salt treatment. (TIF 2714 kb) [file 12870_2016_781_MOESM5_ESM.tif]

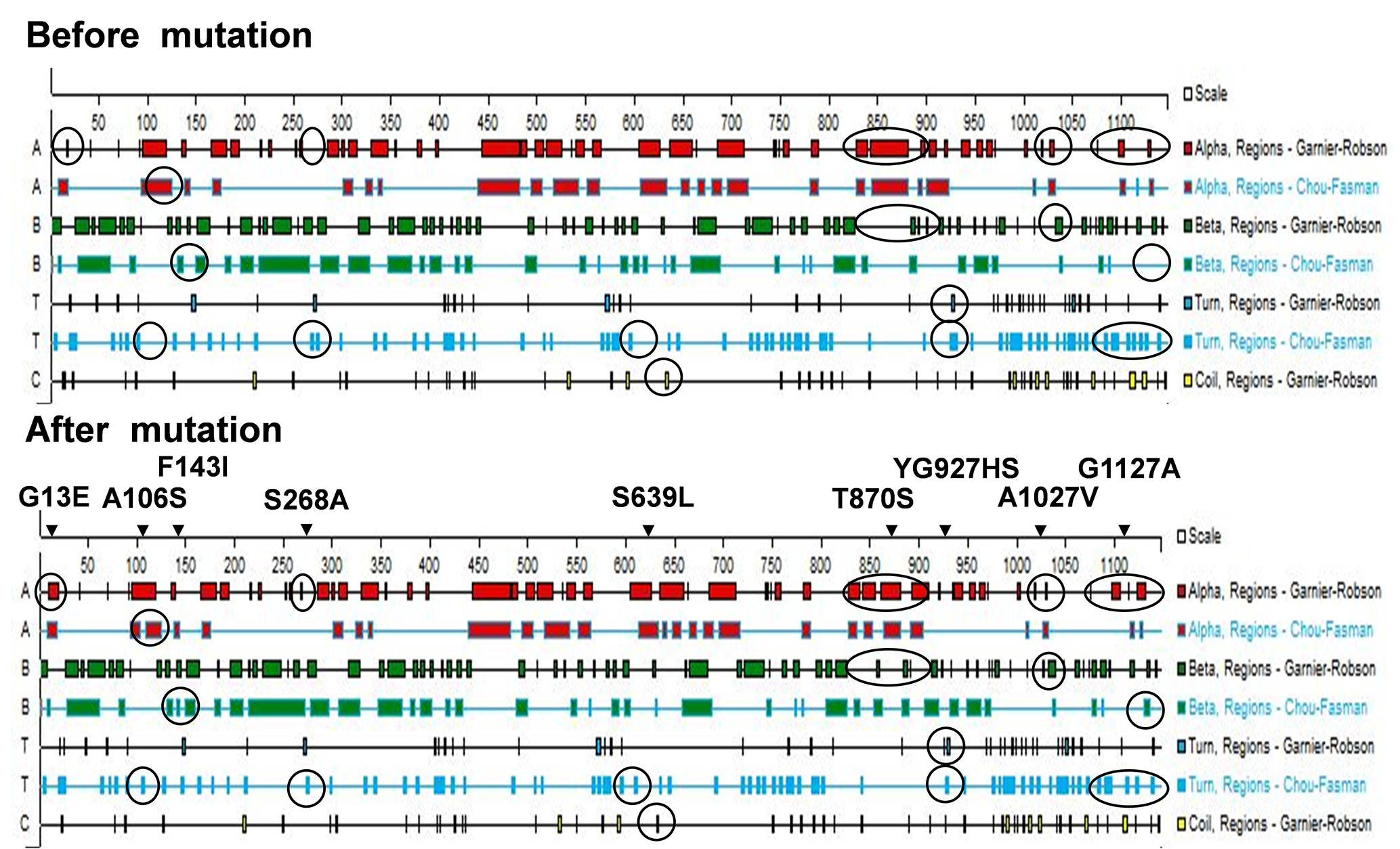

Supplement: Additional file 6: Figure S5. — The predicted secondary structure of AjSOS1 prior to and after site-directed mutagenesis by DNAStar software. (TIF 2480 kb) [file 12870_2016_781_MOESM6_ESM.tif]
