# Supplementary material for: Variation in tissue Na+ content and the activity of SOS1 genes among two species and two related genera of Chrysanthemum
Source: BMC Plant Biol. 2016 Apr 21;16:98. doi: 10.1186/s12870-016-0781-9 (PMC4839091; doi:10.1186/s12870-016-0781-9)
Supplement: Additional file 7: Table S1. — Oligonucleotide sequences used for the site-directed mutagenesis. (DOCX 15 kb) [file 12870_2016_781_MOESM7_ESM.docx]

| Primer | 5′–3′ sequence | Usage |
| --- | --- | --- |
| TB1-F | GCTGAGGGCGGAGGAGGAGGGGG | mutation G13E |
| TB1-R | CCCCCTCCTCCTCCGCCCTCAGC | mutation G13E |
| TB2-F | GAAGAAGGTGGATATAATCCGAGCGATGCGGTGTTA | mutation T26S |
| TB2-R | TAACACCGCATCGCTCGGATTATATCCACCTTCTTC | mutation T26S |
| TB3-F | CTGCTCTTCTTTTTGAAAGTTCATTTTCCATGGAGGTGCAC | mutation A106S |
| TB3-R | GTGCACCTCCATGGAAAATGAACTTTCAAAAAGAAGAGCAG | mutation A106S |
| TB4-F | CTTGGTGCAGCTTTGAAGCTAATTTTTCCATATAACTGGAGTT | mutation F143I |
| TB4-R | AACTCCAGTTATATGGAAAAATTAGCTTCAAAGCTGCACCAAG | mutation F143I |
| TB5-F | GGGTATTGCATTTGGTCTGTTGTCTTATTTGTGGCTTGG | mutation V238L |
| TB5-R | CCAAGCCACAAATAAGACAACAGACCAAATGCAATACCC | mutation V238L |
| TB6-F | GCTACCTTGCTTACTTCACGGCTCAAGAAGGTTTTGATATT | mutation S268A |
| TB6-R | AATATCAAAACCTTCTTGAGCCGTGAAGTAAGCAAGGTAGC | mutation S268A |
| TB7-F | CGTATACTGGATTTCACAAAGCATGAAATGATGAGCAAAGCAC | mutation Y463H |
| TB7-R | GTGCTTTGCTCATCATTTCATGCTTTGTGAAATCCAGTATACG | mutation Y463H |
| TB8-F | GAAGAAGGTAGAATAACCCAGCATACAGCAAATATTCTGATGC | mutation Y549H |
| TB8-R | GCATCAGAATATTTGCTGTATGCTGGGTTATTCTACCTTCTTC | mutation Y549H |
| TB9-F | CCTACTTCACTGTTGAAAGGTTGGAAAGTGCTTGTTACATATCTGC | mutation F609S |
| TB9-R | GCAGATATGTAACAAGCACTTTCCAACCTTTCAACAGTGAAGTAGG | mutation F609S |
| TB10-F | TGACTTCATAGGGAATAGTGAAATAGCTTTGGCAATAATTAATGAGA | mutation S639L |
| TB10-R | TCTCATTAATTATTGCCAAAGCTATTTCACTATTCCCTATGAAGTCA | mutation S639L |
| TB11-F | CCTCAAATCCTTGAGAAAATGTCAATGCATGACTTAAGGACTC | mutation T870S |
| TB11-R | GAGTCCTTAAGTCATGCATTGACATTTTCTCAAGGATTTGAGG | mutation T870S |
| TB12-F | ACCCAGGGTACTTTAGAACTTATTACTACGCCAGCTGCACTATT | mutation A919T |
| TB12-R | AATAGTGCAGCTGGCGTAGTAATAAGTTCTAAAGTACCCTGGGT | mutation A919T |
| TB13-F | GCTGCACTATTCCCTTCACATAGTGACCGAAGTTTCCGAG | mutation YG927HS |
| TB13-R | CTCGGAAACTTCGGTCACTATGTGAAGGGAATAGTGCAGC | mutation YG927HS |
| TB14-F | CATCTTCATTAATATCACATGTTGGCGATAACCCTCCAAG | mutation G982V |
| TB14-R | CTTGGAGGGTTATCGCCAACATGTGATATTAATGAAGATG | mutation G982V |
| TB15-F | CACGGGAACAACTTTTCTGTTCGAGCAATGCAACTAAG | mutation A1027V |
| TB15-R | CTTAGTTGCATTGCTCGAACAGAAAAGTTGTTCCCGTG | mutation A1027V |
| TB16-F | AGCATGATATCTAACGAAGGACAGAGTCCTCGTGCT | mutation H1044Q |
| TB16-R | AGCACGAGGACTCTGTCCTTCGTTAGATATCATGCT | mutation H1044Q |
| TB17-F | AGTCTAATAGCTCCACGTGAAAAGCCAGACCCAAAT | mutation N1109K |
| TB17-R | ATTTGGGTCTGGCTTTTCACGTGGAGCTATTAGACT | mutation N1109K |
| TB18-F | GAGCGATGAGTCTGGAGCTGAGGATGAACACATTG | mutation G1127A |
| TB18-R | CAATGTGTTCATCCTCAGCTCCAGACTCATCGCTC | mutation G1127A |
| TB19-F | CCACCCTCATACTGCAATAGGGAATGATAATGATGTTGAACC | mutation E512G |
| TB19-R | GTTCAACATCATTATCATTCCCTATTGCAGTATGAGGGTGG | mutation E512G |
